# Technical aspects of SBRT for therapy-refractory ventricular tachycardia: a systematic review for radiation oncologists

**DOI:** 10.1186/s13014-025-02704-w

**Published:** 2025-08-29

**Authors:** Alicia Greiner, Lukas Grajewski, Maximilian Römer, Klaus Pietschmann, Georg Wurschi

**Affiliations:** Department of Radiotherapy and Radiation Oncology, Jena University Hospital, Jena, Germany

**Keywords:** Ventricular tachycardia, Stereotactic body radiation, Technical aspects, Safety, Efficacy

## Abstract

**Background:**

Ventricular tachycardia (VT) is a potentially life-threatening arrhythmia, that can lead to sudden cardiac death. While conventional treatments include antiarrhythmic drugs, catheter ablation, and ICD implantation, recent studies suggest that stereotactic body radiotherapy (SBRT) offers a non-invasive alternative for the treatment of VT. The objective of this systematic review is to summarize the current evidence on SBRT for refractory VT from a radiotherapeutic and technical perspective and to assess its safety and effectiveness.

**Methods:**

he systematic search was conducted according to the PRISMA guidelines, using the four databases PubMed, Cochrane, Scopus, and Web of Science. After screening, 15 publications were included and analyzed in this review.

**Results:**

A total of 15 studies were identified in the literature, describing 173 patients. SBRT was delivered as a single fraction using standard LINAC- or CyberKnife-devices with varying isodose prescriptions, motion management, and imaging guidance, highlighting the need for standardized protocols. SBRT consistently reduced VT burden across these studies, with reduction rates ranging from 50 to 99%, and improved quality of life in some patients. However, VT recurrences have also been described.

**Conclusion:**

Delivery of SBRT for VT is similar to that for malignant diseases but requires specialized imaging and mapping procedures to ensure precise delivery of radiation to small ectopic areas within the beating heart. SBRT seems safe and effective in reducing VT in first clinical trials, though it is not yet a routine treatment. Further controlled trials with standardized treatments and endpoints are needed for confirmation.

**Clinical trial number:**

This study does not involve a clinical trial; therefore, a clinical trial number is not applicable.

**Graphical Abstract:**

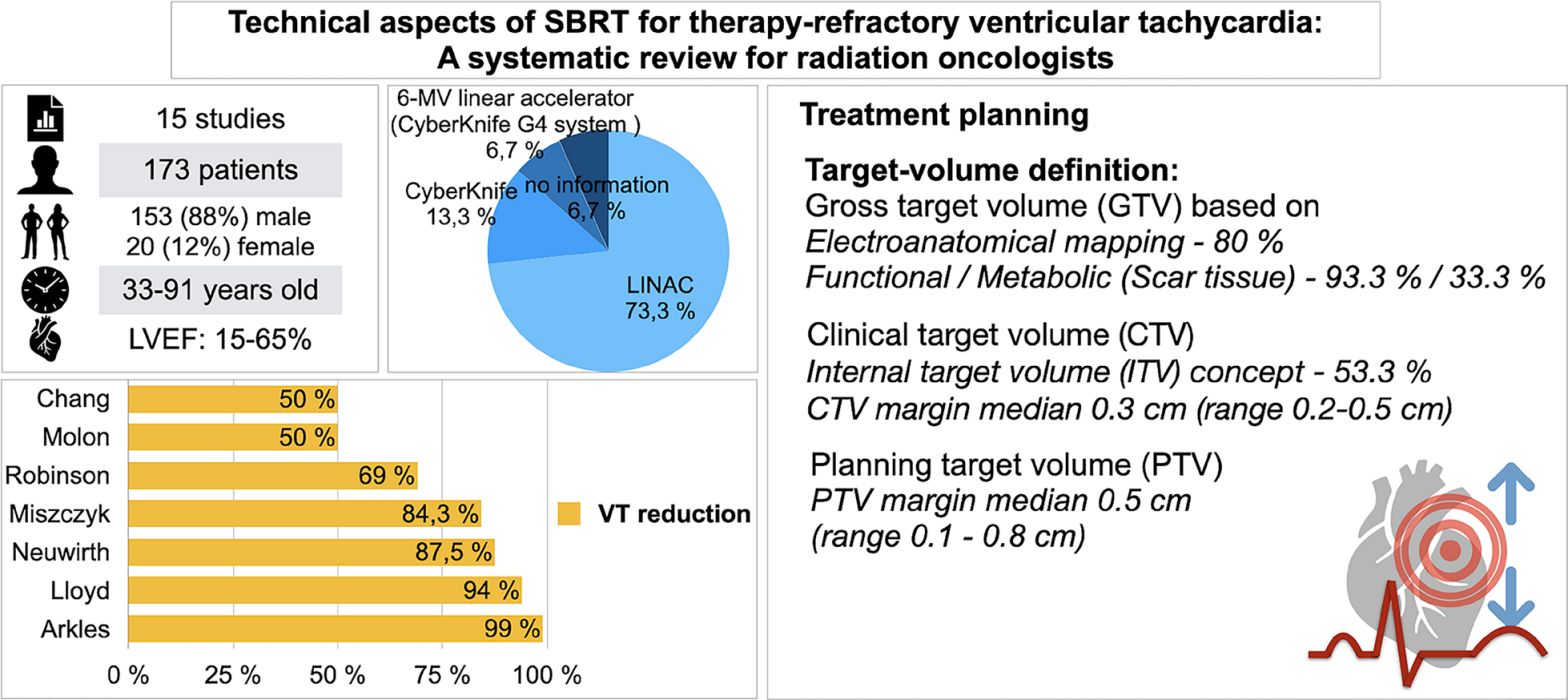

**Supplementary Information:**

The online version contains supplementary material available at 10.1186/s13014-025-02704-w.

## Introduction

Ventricular tachycardia (VT) is a wide complex tachycardia, which is emerging from the ventricle. It is defined as three or more sequential beats at a rate exceeding 100 beats per minute. This potentially life-threatening arrhythmia is a major contributor to sudden cardiac death in patients with ischemic and non-ischemic cardiomyopathy. VT is classified in monomorphic and polymorphic patterns, depending on the underlying disease and localization of the ectopy. Monomorphic VT is usually seen in cardiomyopathy due to localized scar-related reentry, which may lead to ventricular fibrillation, cardiac arrest or death [1]. Polymorphic VT may origin from different ectopic areas through myocardial ischemia [2]. The prevailing treatment is primarily depending on the underlying structural heart disease. In case of transient myocardial ischemia, coronary angiography might be an effective treatment. Apart from that, symptomatic antiarrhythmic treatment, including the acute administration of β-blockers, amiodarone or sedation, which is generally required to suppress the sympathetic tone, are applied. Interventional treatment modalities include catheter ablation (CA) of ectopic areas, implantable cardioverter-defibrillator (ICD) and sympathetic blockade by left cardiac sympathetic denervation to modify the autonomic tone [3, 4]. Despite the effectiveness of ICDs in terminating VT and the reduction in ICD shocks through radiofrequency CA, long-term recurrence rates prevail at over 50% beyond several trials in patients with structural heart disease [5].

A newer innovative therapy method is the non-invasive ablation of ectopic areas by stereotactic body radiation (SBRT). SBRT is an advanced radiotherapy technique, mainly used for local ablative treatment of low-volume tumor mass, such as early-stage primary and various cerebral, bone, liver or lung metastasis of oligometastatic disease [6]. Opposed to conventional radiotherapy, which involves the application of conventional or moderately hypofractionated doses (single fraction dose, ≤ 4 Gy) over several weeks, SBRT delivers a higher, mostly locally ablative, dose in one to five treatment sessions. Cardiac SBRT protocols for VT demonstrate variability in dose prescription and delivery techniques; for example, certain systems such as CyberKnife^®^ apply dose prescriptions based on specific isodose levels, resulting in an inherently inhomogeneous dose distribution. However, other SBRT protocols may use more homogeneous dose prescriptions depending on institutional practices and treatment planning approaches [5]. For this reason, this technique and the devices used for it are subject to special requirements regarding precision, a steep dose gradient, and adequate imaging guidance or motion control.

Recent studies have also demonstrated the efficacy of SBRT in the management of VTs. However, treatment specifications are not standardized and radiotherapeutic aspects have not been addressed up to now [7, 8].

The aim of this review was to specifically summarize the current evidence regarding SBRT for refractory VT from a radiotherapeutic and technical perspective and to assess its safety and effectiveness systematically.

## Methods

### Criteria for including and excluding studies in the review

Inclusion and exclusion criteria are listed in Table 1 based on a PICO model.

The selection criteria included prospective and retrospective trials on SBRT of refractory VT.

Eligible studies had to represent efficacy and/or safety endpoints. For efficacy they needed to address at least one of the following endpoints: reduction of VTs, mortality or quality of life (QoL). For safety assessment they needed to represent toxicity or adverse events (AEs). Radiotherapy treatment specifications, such as dose prescription and treatment device, had to be sufficiently reported. To reduce bias, standardized studies were selected and evaluated.

Criteria for rejecting studies were patients under 18 years, studies on animals, gray literature, other publication types than primary investigation/report (e.g., comments, letters, abstracts) or if there was no full text available.

Language restrictions were made to English and German.

**Table 1:** PICO criteria for including and excluding studies in the review

PICO	Inclusion criteria	Exclusion criteria
Patient	Patients with refractory ventricular tachycardia	Patients < 18 years
Intervention	single fraction or fractionated (2–5) SBRT, STAR, SABR to the heart	
Comparison	Any	
Outcome	Efficacy (Reduction of VTs, Mortality, Quality of life)	
	Safety (Toxicity and adverse events (CTCAE))	
	Treatment specifications	
Others	Language: German and English Full publication	Gray literature (conference articles, abstracts, letters, unpublished literature, etc.) Full text not available in German or English

### Study selection

The systematic research was conducted in accordance with the PRISMA guideline [9] using four databases (PubMed, Cochrane, Scopus, and Web of Science) first in November 2023. A further screening for review and update was carried out in January 2025. For each of these databases, a complex search strategy was developed, consisting of a combination of MeSH terms, keywords, and text words in various spellings related to ventricular tachycardia and stereotactic body radiation, linked to the keywords SBRT, STAR or SABR. The full search string is enclosed in detail within the Appendix A1.

Following the removal of duplicates using EndNote 20, the remaining records were subjected to independent screening by two reviewers (AG, LG) in a two-step process. In the initial phase, titles were examined and excluded if their content was clearly unrelated to the subject of SBRT for refractory ventricular tachycardia. Excluded records were thematically categorized, for example as studies addressing VT treatment without SBRT, SBRT applied to non-cardiac targets, cardiac radiotherapy not aimed at VT such as metastases or sarcomas, or other types of arrhythmias such as atrial fibrillation. In the subsequent phase, the abstracts of the remaining records were subjected to a more meticulous independent evaluation. Studies were excluded if they did not meet the predefined criteria, including studies not involving VT or SBRT, studies focusing on other populations or indications, non-original research such as narrative reviews or case reports without primary data, animal studies, and articles not published in English or German. A total of 5901 records were excluded at the title and abstract level, with the majority of these records falling into the specified categories. Despite the absence of a structured automation tool for pre-filtering, the dual independent review process ensured consistency and rigor. Disagreements were resolved by consensus.

This process yielded 26 studies that were eligible for full-text screening. Where abstracts did not provide sufficient information, full texts were retrieved at an earlier stage. All full texts were independently reviewed by both reviewers, and bibliographies of included articles were manually screened for further relevant studies. Studies were included if they offered a comprehensive description of the use of SBRT in VT patients, including baseline characteristics and outcome measures.

**Fig. 1:**
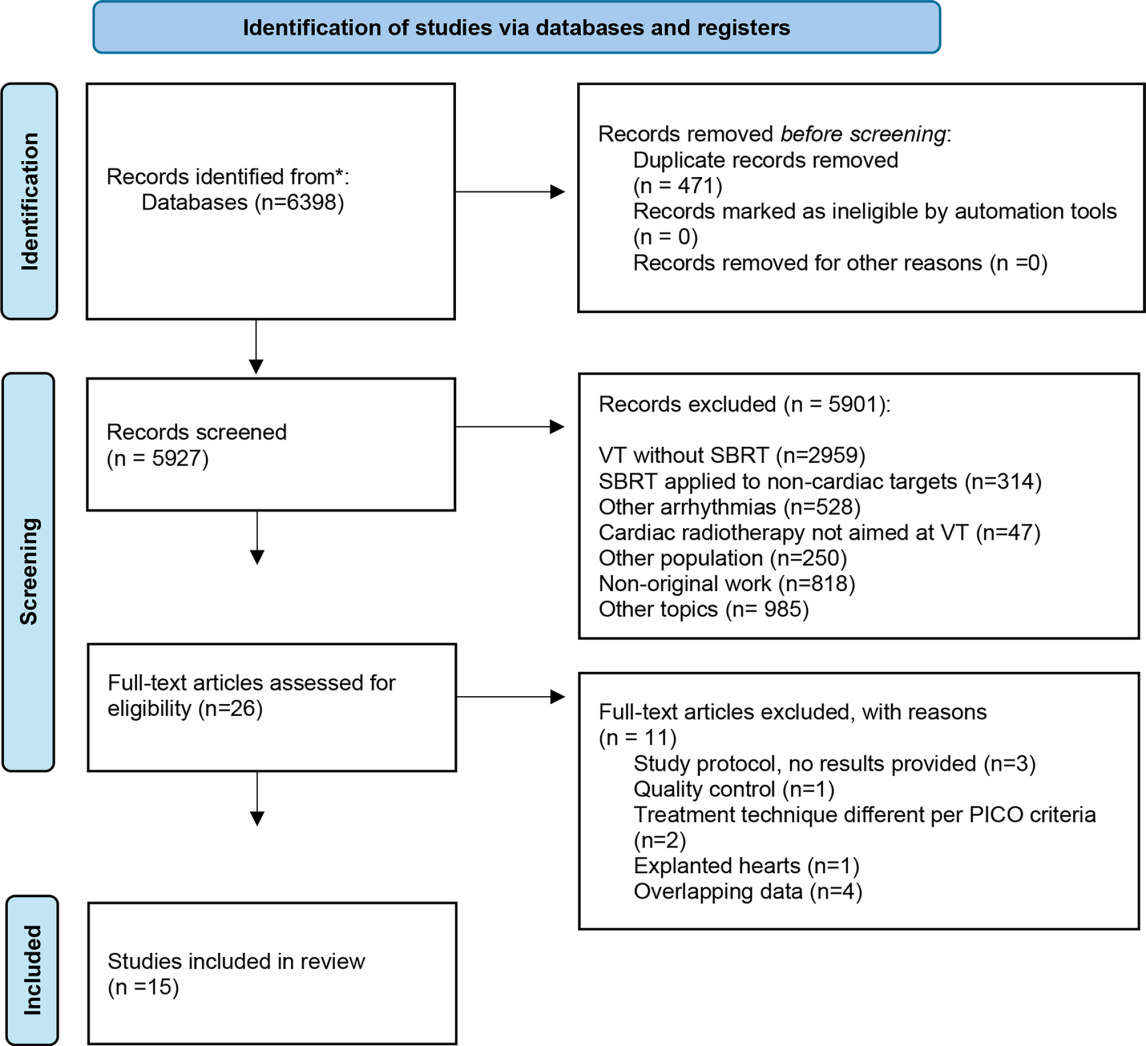
Preferred reporting items for systematic reviews flow chart displaying the study selection process

Figure 1 outlines the selection process, and Appendix A2 lists all full-text exclusions. Figure artwork was created using Keynote, Version 14.3 (Apple Inc.).

### Assessment of risk of bias and methodological quality

All characteristics were assessed by two independent reviewers (AG, LG). In case of ambiguities, a third reviewer (GW) was included, and an agreement was made by discussion.

### Risk of bias

The risk of bias was analyzed with the Cochrane Risk of Bias tool, for systematic reviews, ROBINS-I [10]. The studies were also rated with the Oxford criteria.

### Data extraction

Data extraction was performed by two independent reviewers (AG, LG), controlled and discussed with another reviewer (GW).

Extraction was limited to data from primary literature and other sources, which provided a comprehensive description of the study, meeting the inclusion criteria.

Accounting for the significant overlap in the studies included within the systematic reviews, only data from individual studies was extracted. The extraction was restricted to primary literature and other sources that provided comprehensive study details and met the inclusion criteria.

## Results

The systematic search revealed 6398 results. At first, duplicates were removed leaving 5927 studies. After screening of title and abstract, 26 studies remained to complete review. Finally, 15 publications were analyzed in this review, including 13 prospective and 2 retrospective studies. Overall, 173 patients were described in 15 publications.

### Characteristics of included studies

Of the 173 patients included in the studies, 153 (88%) were male and 20 (12%) were female, with ages ranging from 33 to 91 years. A detailed overview about the study characteristics is shown in Table 2.

**Table 2:** Study characteristics reported in the studies

Year	Author	Title	Type of study	Population (*N*)	Underwent treatment (*N*/%)	Endpoints		
							Primary endpoint	Secundary endpoint
2023	Amino et al.	Interim Report of a Japanese Phase II Trial for Cardiac Stereotactic Body Radiotherapy in Refractory Ventricular Tachycardia Focus on Target Determination	prospective Phase II interventional single-arm open-label asymmetric single-center study	3	3 / 100	(1) safety (2) efficacy	safety: adverse events and arrhythmia suppression (including ICD shocks, antitachycardia pacing [ATP] events, and manual direct current shocks)	efficacy: reduction in arrhythmic events and improvement in cardiac function, assessed by non-invasive parameters like high-resolution ECG, myocardial imaging, and reduction in premature ventricular contractions and non-sustained VT frequency
2022	Aras et al.	Stereotactic body radioablation therapy as an immediate and early term antiarrhythmic palliative therapeutic choice in patients with refractory ventricular tachycardia	prospective case series, single center	8	8 / 100	efficacy	efficacy: any reduction in the number of ICD treatments for VT episodes and/or any reduction in VT episodes comparing the 6 months before and after treatment	not reported
2024	Arkles et al.	One-year outcomes after stereotactic body radiotherapy for refractory ventricular tachycardia	prospective cohort study	15	14 / 93.3	(1) efficacy (2) safety	efficacy: reduction in treated VT episodes, ICD shocks, and antitachycardia pacing sequences following SBRT treatment	safety and heart failure status, need for antiarrhythmic drugs (AADs)
2021	Carbucicchio et al.	Stereotactic radioablation for the treatment of ventricular tachycardia: preliminary data and insights from the STRA-MI-VT phase Ib/II study	spontaneous, prospective, single-arm, phase Ib/II single-center study; STRA-MI-VT	8	7 / 87.5	(1) safety (2) efficacy	safety: safety of SBRT during the early first month phase after SBRT and at the 3-, 6-, and 12-month follow-up (FU) efficacy: represented by the total number of VT/ventricular fibrillation (VF) episodes detected by the ICD at 3, 6, and 12 months, compared to the 3-month period preceding SBRT: (1) Number of VT/VF episodes causing antitachycardia pacing (ATP) (2) number of VT/VF episodes causing ICD shock 3. total number of ICD shocks 4. number of VT episodes below the tachycardia detection interval (TDI)	evaluated at 3, 6, and 12 months: 1.Global mortality (at 12 months) 2. variations in quality of life (QoL) 3. using the SF-36 Health Questionnaire 4. changes in cardiac function assessed by LV EF on echocar- diographic examination.
2023	Chang et al.	Short-term and long-term effects of noninvasive cardiac radioablation for ventricular tachycardia: A single-center case series	prospective case series	6	6 / 100	efficacy	efficacy of radioablation in reducing VT episodes	long-term safety and clinical outcomes
2021	Chin et al.	Non-invasive stereotactic body radiation therapy for refractory ventricular arrhythmias: an institutional experience	retrospective, single-center	8	8 / 100	efficacy	efficacy: reduction in ICD therapies compared to pre-treatment levels​	efficiacy: reduction in VT episodes and improvement in quality of life​ safety:
2020	Gianni et al.	Stereotactic arrhythmia radioablation for refractory scar-related ventricular tachycardia	pilot prospective, single arm, two-center feasibility study	6	5 / 83.3	efficacy	freedom from ventricular arrhythmias post-procedure.	reduction in antiarrhythmic drug (AAD) usage.
2024	Hašková et al.	Efficacy and Safety of Stereotactic Radiotherapy in Patients With Recurrent Ventricular Tachycardias	observational cohort study	efficacy cohort: 17 safety cohort: 36	efficacy cohort: 17 / 100 safety cohort: 36 / 100	1. efficacy 2. safety	reduction in the burden of ICD therapies (shocks) after STAR.	long-term safety and adverse effects associated with STAR
2023	Krug et al.	Radiosurgery for ventricular tachycardia (RAVENTA): interim analysis of a multicenter multiplatform feasibility trial	prospective multicenter multiplatform RAVENTA study	5	5 / 100	1. safety 2. efficacy	30- day postprocedural safety	evaluated within the first year of follow up: 1. rate of VT episodes and ICD interventions 2. use of antiarrhythmic medication 3. toxicity 4. quality of life 5. overall survival
2020	Lloyd et al.	Clinical experience of stereotactic body radiation for refractory ventricular tachycardia in advanced heart failure patients	single-center retrospective study	10	Aug-80	efficacy	total number of seconds of detected VT before and after SBRT.	number of ATP sequences and ICD shocks recorded before and after treatment
2023	Miszczyk et al.	Stereotactic management of arrhythmia- radiosurgery in treatment of ventricular tachycardia (SMART-VT). Results of a prospective safety trial	prospective single-arm safety trial	11	11 / 100	1. safety 2. efficacy	safety: sate of treatment-related grade 3 serious adverse events (AEs) within the first 90 days	efficacy: reduction of VT burden, changes in QoL safety: cardiac injury markers, late toxicity
2022	Molon et al.	Stereotactic ablative radiotherapy in patients with refractory ventricular tachyarrhythmia	prospective mono-institutional pilot study	6	6 / 100	1. efficacy 2. safety	efficacy: reduction in ICD shocks after SABR treatment	safety of the procedure
2019	Neuwirth et al.	Stereotactic radiosurgery for ablation of ventricular tachycardia	prospective Phase I/II trial/ case series	10	10 / 100	efficacy	efficacy: change in the frequency of sustained VT episodes	efficacy: occurrences of electrical storm, antitachycardia pacing, shock, time to death, and radiation-induced events
2019	Robinson et al.	Phase I/II Trial of Electrophysiology-Guided Noninvasive Cardiac Radioablation for Ventricular Tachycardia	prospective phase I/II trial	19	19 / 100	efficacy	efficacy: any reduction in VT episodes or PVC burden	efficacy: 50% and 95% reduction in VT episodes or PVC burden, elimination of ICD shocks, improvement in left ventricular ejection fraction
2023	van der Ree et al.	Non-invasive stereotactic arrhythmia radiotherapy for ventricular tachycardia: results of the prospective STARNL-1 trial	prospective monocentre single-arm pre-post intervention study	6	6 / 100	1. efficacy 2. safety	efficacy: reduction in the arrhythmic burden after the SABR treatment, reduction in the number of treated VT-episodes by ≥50% at the end of follow-up	safety: acute and late toxicity, the all-cause, and cause-specific mortality, changes in left ventricular ejection fraction, pulmonary function, and quality of life measures

Across the studies, ischemic cardiomyopathy (ICM) was observed in more than 50% of patients in most cohorts. Notable exceptions included Aras et al. [11] (ICM: 1/4 [25%]) and Hasková et al. [12], where ICM was present in 29% of the efficacy cohort and 56% of the safety cohort. The proportion of patients with prior CA varied considerably, ranging from 33% [13] to 100% [14–16]. The studies that reported New York Heart Association (NYHA) score (5/15), showed that the full range from I to IV was represented, with class II and III being the most frequently reported. Among the 15 studies, 14 reported predominantly reduced left ventricle ejection function (LVEF); within these, Amino et al. [13] included 1 patient with preserved function (65%). The baseline criteria are presented in detail in Table 3.

**Table 3:** Summary of baseline characteristics

Author	Age (years), mean / range	Population (*N*)	Sex (*N* / %)	LVEF (%), mean	LVEF (%), median / range	ICM (%)	previous CA (%)	AADs (%)	NYHA score, range
Amino et al.	not reported / 60 - 91	3	m 1 / 33 f 2 / 67	not reported	not reported / 20 - 65	66.6	33.3	100	II-IV
Aras et al.	58 ± 14 / 33 - 85	8	m 8 / 100 f 0 / 0	24 ± 5	not reported	25	87.5	100	II-III
Arkles et al.	65.0 ± 7.8 / not reported	15	m 13 / 87 f 2 / 13	30.2 ± 3.6	not reported	46	60	100	not reported
Carbucicchio et al.	70 ± 7 / 59 - 81	8	m 8 / 100 f 0 / 0	27 ± 10	not reported	42.9	71.4	100	not reported
Chang et al.	72 ± 7.4 / 64 - 72	6	m 4 / 67 f 2 / 33	34 ± 12.3	not reported	not reported	67	100	III-IV
Chin et al.	75 ± 7.3 / 65 - 86	8	m 8 / 100 f 0 / 0	21 ± 7	not reported	50	75	100	not reported
Gianni et al.	63 ± 12 / 45 - 76	6	m 6 / 100 f 0 / 0	34 ± 15	not reported	80	100	100	not reported
Hašková et al.	efficacy cohort: 65 ± 11 / not reported safety cohort: 66 ± 10 / not reported	efficacy cohort: 17 safety cohort: 36	efficacy cohort: m 15 / 88 f 2 / 12 safety cohort: m 33 / 92 f 3 / 8	efficacy cohort: 30 ± 10 safety cohort: 31 ± 9	not reported	efficacy cohort: 29 safety cohort: 56	100	efficacy cohort: 76 safety cohort: 74	not reported
Krug et al.	not reported / 49 - 74	5	m 4 / 80 f 1 / 20	not reported	not reported / 20 - 45	40	80	100	not reported
Lloyd et al.	61 / 50 - 78	10	m 7 / 70 f 3 / 30	not reported	not reported	40	100	100	not reported
Miszczyk et al.	67 / 45 - 72	11	m 10 / 91 f 1 / 9	not reported	27 / 20 - 40	81.8	54.5	100	I-III
Molon et al.	not reported / 61 - 85	6	m 5 / 83 f 1 / 17	not reported	not reported / 20 - 42	not reported	not reported	not reported	II-IV
Neuwirth et al.	66 / 61 - 78	10	m 9 / 90 f 1 / 10	26.5 ± 3.2	not reported	80	100	not reported	not reported
Robinson et al.	66 / 49 - 81	19	m 17 / 90 f 2 / 10	not reported	25 / 15 - 58	57.9	84.2	not reported	not reported
van der Ree et al.	73 / 54 - 83	6	m 6 / 100 f 0 / 0	not reported	38 / not reported	100	100	100	not reported

The targeting and treatment characteristics presented significant variability across the trials. A comprehensive overview is provided in Table 4.

**Table 4:** Treatment planning and characteristics of the reported studies

Author	total adsorbed dose / single fraction dose (Gy)	Mapping software	GTV (cc), mean / median / range	CTV (cc), mean / median / range	PTV (cc), mean / median / range	Prescription isodose line (%), specified / mean / median / range	PTV coverage (%)	Treatment device	Radiation technique	Radiation planning software	Beam on time (min), mean / median / range	Treatment duration (min), mean / median / range	Image guidance
Amino et al.	25 / 25	not reported	not reported	not reported / not reported / 8.3 - 24.2	not reported / not reported / 49.7 - 96.4	not reported	not reported	linear accelerator (TrueBeam STx; Varian Medical Systems Inc.)	VMAT	Eclipse ver. 13.7; Varian Medical Systems Inc., Palo Alto, CA, USA	not reported / not reported / 2.6 - 5.2	not reported / not reported / 34 - 48	CBCT
Aras et al.	25 / 25	CARTO^®^ 3 three-dimensional electroanatomic mapping system (Biosense Webster, Diamond Bar, CA, USA)	not reported	not reported	not reported / 157.4 / 70.5 - 272.7	95 / not reported / not reported / not reported	not reported	linear accelerator (TrueBeam^®^, Varian Medical)	not reported	EclipseTM, Varian Medical	not reported / 5.6 / 3.6 - 7.45	not reported	CBCT
Arkles et al.	25 / 25	ADAS Medical (Galgo Medical SL, Barcelona, Spain); MUSIC (Institut Hospitalo-Universitaire l’Institut de Rythmologie et Modélisa- tion Cardiaque, Université de Bordeaux, Bordeaux, France, and Inria Sophia Antipolis, Bordeaux, France)	26.8 ± 9.8 / not reported / not reported	not reported	84.9 ± 24.4 / not reported / not reported	not reported	not reported	linear accelerator (Varian TrueBeam Linac)	not reported	Varian Eclipse software	not reported / 3.45 / 2.62 - 4.58	not reported	CBCT
Carbucicchio et al.	25 / 25	CARTO system (Biosense Webster, Inc.)	not reported	39 ± 17 / not reported / not reported	183 ± 53 / not reported / not reported	95 / not reported / not reported / not reported	not reported / 96 ± 1 / not reported / not reported	linear accelerator Varian Trilogy (Varian Medical Systems)	VMAT	Eclipse Varian Treat- ment Planning System (Varian Medical System)	not reported	31 ± 6 / not reported / not reported	CBCT
Chang et al.	25 / 25	CARTO 3 electroanatomical mapping system (Biosense Webster, Diamond Bar, CA)	20.2 ± 29 / not reported / not reported	not reported	83.9 ± 85.9 / not reported / not reported	not reported	not reported / not reported / not reported / 90 - 95	linear accelerator True-Beam (Varian Medical Systems)	VMAT	Eclipse system (Varian Medical Systems, Palo Alto, CA)	7.8 ± 6.6 / not reported / not reported	21.2 ± 28.6 / not reported / not reported	CBCT
Chin et al.	Pt 1: 15 / 15 Pt 2,3: 20 / 20 Pt 4-8: 25 / 25	not reported	34.2 ± 19.3 / not reported / not reported	not reported	121.4 ± 50.4 / not reported / not reported	not reported	not reported	linear accelerator Novalis Tx Medical (Brainlab, Inc., Westchester, IL)	VMAT	MIM software (MIM Inc., Beachwood, OH)	not reported	18.2 ± 6.0 / not reported / not reported	CBCT
Gianni et al.	25 / 25	not reported	not reported	not reported	143 ± 50 / not reported / not reported	not reported / 77 / not reported / 74 - 80	not reported / 82 ± 17 / not reported / not reported	6-MV linear accelerator (CyberKnife G4 system with an Iris variable aperture collimator)	not reported	CardioPlan software (CyberHeart, Portola Valley, CA, USA; MultiPlan Treatment Planning Software for Cyberknife (Accuray, Sunnyvale, CA, USA)	not reported	not reported	X-ray guided
Hašková et al.	25 / 25	not reported	not reported	not reported	not reported / 39.4 / 12.6 - 90.5	not reported / not reported / 78 / 66 - 84	95 / not reported / not reported / not reported	CyberKnife radiosurgery system (Accuray, Inc)	Robotic	MultiPlan treatment planning system with sequential dose optimization	not reported	not reported / 58 / 42 - 82	not reported
Krug et al.	25 / 25	CARDIO-RT (University of Lübeck, Germany)	not reported	not reported / 8.1 / 6.0 - 34.4	not reported / 69.6 / 43.4 - 80.7	95 / not reported / not reported / not reported	not reported	not reported	DCA (*n*=2), IMAT (*n*=2), Robotic (*n*=1)	not reported	not reported	not reported / 20 / 9 - 61	CBCT (*n*=4), stereoscopic X-ray (*n*=1)
Lloyd et al.	25 / 25	EnSite Precision (Abbott, Abbott Park, IL); CARTO (Biosense Webster, Diamond Bar, CA)	not reported	not reported	81.4 ± 56 / not reported / not reported	not reported	95 / not reported / not reported / not reported	linear accelerators (Varian Trubeam)	VMAT	Eclipse treatment planning software (Var- ian, Palo Alto, CA)	not reported	30 / not reported / not reported	CBCT, kV imaging
Miszczyk et al.	25 / 25	CARDIO-RT software	not reported	not reported	not reported / 73 / 18.6 - 111.3	not reported	not reported	linear accelerator (Varian EDGETM)	VMAT	not reported	not reported / 13.43 / 9.42 - 18.9	not reported / 43.9 / 31.57 - 101.78	2D kV-kV imaging, respiratory-gated CBCT
Molon et al.	25 / 25	CardioInsightTM— Medtronic, (Minneapolis, MN, USA)	not reported	not reported	not reported	not reported	not reported	linear accelerator TrueBeam Linac (Varian Medical Systems, Palo Alto, CA, USA)	VMAT	Eclipse v. 16.1.4.4	not reported	not reported	not reported
Neuwirth et al.	25 / 25	CARTO; (Biosense-Webster, Israel)	not reported	not reported	22.2 / not reported / 14.2 - 29.6	not reported / 80 / not reported / 66–84	not reported	CyberKnife radiosurgery system	Robotic	MultiPlan treatment plan- ning system (Accuray, Inc., Sunnyvale, CA, USA)	not reported	68 / not reported / 45–80	control X-ray
Robinson et al.	25 / 25	not reported	not reported / 25.4 / 6.4 - 88.6	not reported	not reported / 98.9 / 60.9 - 298.8	not reported	not reported	Linear accelerator (Varian TrueBeam, Varian Edge)	VMAT or multiple-plane, fixed pattern (fixed-field)	Pinnacle, Phillips, Amsterdam, or Eclipse, Varian Medical Systems, Palo Alto	not reported / 15.3 / 5.4 - 32.3	not reported	CBCT
van der Ree et al.	25 / 25	not reported	not reported	not reported / 46 / 15 - 87	not reported / 187 / 93 - 372	not reported	95 / not reported / not reported / not reported	linear accelerator Agility™ (Elekta, Sweden)	VMAT	not reported	not reported / 4.6 / 3.6 - 5.2	not reported	not reported

Additionally, the duration of the follow-up period demonstrated significant variation among the studies, with a median ranging from 6 months [11, 14, 17] to 28 months [15].

### Excluded studies

12 studies were excluded after full-text screening, 3 of them because they only showed a study protocol, one of them was a study researching on explanted hearts, in one of the records they were working with dose escalation and 5 studies showed off as overlapping data from large trials.

### Risk of bias in included studies

The methodical quality of the studies was analyzed with the Cochrane Risk of Bias tool ROBINS I [10]. A representation of this is provided in Appendix A3. The studies were also rated according to the Oxford criteria, which allowed the evidence level to be determined.

### Treatment specifications

SBRT was delivered within a single-fraction dose of 25 Gy. However, the prescribed isodose line varied across the trials. The prescription isodose line was only documented in 6 of the 15 studies, with 3/6 employing a prescription to the 95% isodose line [11, 17, 18]. Other trials (3/6) reported a median prescription to 77% − 80% (range 66–84%) [12, 16, 19]. The planning target volume (PTV) coverage was also mentioned in 6 studies only. In 5 of them reaching at least 95% [12, 14, 17, 20, 21] while one of them involved a single patient reaching 90% [15]. Only 1 trial reported lower coverage (82%, range 61–95%) [19].

The treatment was delivered with up-to-date linear accelerators (LINACS) (73.3%) or CyberKnife^®^ (13.3%) devices. In the study by Gianni et al. [19] the 6-MV linear accelerator (CyberKnife G4 system with Iris variable aperture collimator) was used, which includes both systems and 1 study [18] did not report the device used. Most studies (60%) used volumetric modulated arc therapy (VMAT) as delivery technique. In the study by Krug et al. [18] different radiation techniques were used; dynamic conformal arc (DCA) in 2 patients, intensity-modulated arc therapy (IMAT) in 2 patients and robotic in 1 patient. The studies with the CyberKnife devices [12, 16] used a robotic delivery technique, and 3 studies [11, 19, 22] did not report.

Patients were mostly treated in supine position. As general immobilization techniques, some studies documented the use of vacuum devices thermoplastic masks or foam cushions [13, 22, 23, 25]. Various motion management strategies have been reported to enhance the precision of SBRT delivery. These include abdominal compression [13, 22, 25] to minimize respiratory-induced tumor motion and 4D-CT-based planning [20, 22, 23].

Additionally, respiratory gating techniques were selectively implemented across studies to ensure the stability of target movement. As indicated by the RAVENTA study [18], Deep Inspiration Breath-Hold (DIBH) was employed in 1/5 of patients. This approach was adopted in 10/11 patients in the SMART-VT study [15] and 2/6 patients in the study conducted by Chang et al. [20] Continuous Positive Airway Pressure (CPAP) was utilized in 1 patient in the study by Chang [20]. Neuwirth et al. [16] and Hašková et al. [12] reported the utilization of breath-hold CT with intravenous contrast enhancement for their entire patient population. The use of motion management techniques has been documented in those studies with differences in consistency in individual patients.

Treatment planning was carried out with established radiotherapy planning software in all cases. Target volume definition was either based on functional imaging, identifying (hypometabolic) scar tissue by PET/CT or scintigraphy (33.3%), or on electroanatomic mapping (EAM) procedures (80%), identifying arrhythmogenic areas. Mapping of ectopic areas was reported for 9/15 studies. Most studies (60%) have utilized an internal target volume (ITV) approach to account for cardiorespiratory motion during treatment planning; however, they have differed in their specific methodologies and margin definitions.

An additional PTV margin of 2–5 mm was usually applied. Nearby organs-at-risk usually comprised the esophagus, lungs, heart and stomach, where common normal-tissue constraints for SBRT were applied. Additionally, relevant anatomic structures of the hearth, including the heart valves and coronary arteries, were partly contoured.

The use of fiducial markers like electrodes of cardiac devices has been implemented to improve target localization and tracking [14, 16, 18, 19]. ECG monitoring during the treatment was only mentioned in 3 of the studies [15, 21, 23]. In the study by Aras et al. [11] an average phase CT was created to evaluate cardiac and respiratory movements.

Pretreatment image guidance was always required, while 66.7% of the studies utilizing cone beam CT (CBCT), 26.7% mentioned X-Ray guidance and 13.3% were also using kV-imaging. The mean overall treatment was reported to be about 20–30 min (range 9–102 min).

Post-treatment monitoring protocols varied across the studies but generally included a combination of clinical assessments, imaging, and device interrogations. Most studies (86.7%) implemented in-patient monitoring for 24 h hours, subsequent repeated 12-lead ECG controls, ICD interrogations, or echocardiography at predefined intervals. Only 1 study [11] documented the prescription of oral anticoagulation as a post treatment measure. No in-patient treatments were routinely required after administration of SBRT.

A detailed overview of treatment planning and characteristics is represented in Table 4 and a thorough exposition of motion management, treatment planning, and post-treatment measures can be found in the Appendix A4.

### Efficacy of cardiac stereotactic body radiation

#### VT-Reduction

The included studies delivered a variety of results and reported ranging degrees of reduction in VT burden after SBRT. In certain cohorts, individual patients experienced complete suppression of VT episodes, specifically during early follow-up periods [20, 22, 24]. More commonly, there were partial reductions in VT burden, often accompanied by decreases in ICD therapies and the use of antiarrhythmic medication [14–16, 21, 25]. A number of studies have also noted VT recurrence beyond the initial blanking period. In some cases, this has required repeat CA or readmission of medication for certain patients [12, 17, 19, 23]. A number of cohorts have reported outcomes that are mixed or non-significant, reflecting heterogeneity in patient response and follow-up durations [19, 23].

**Fig. 2:**
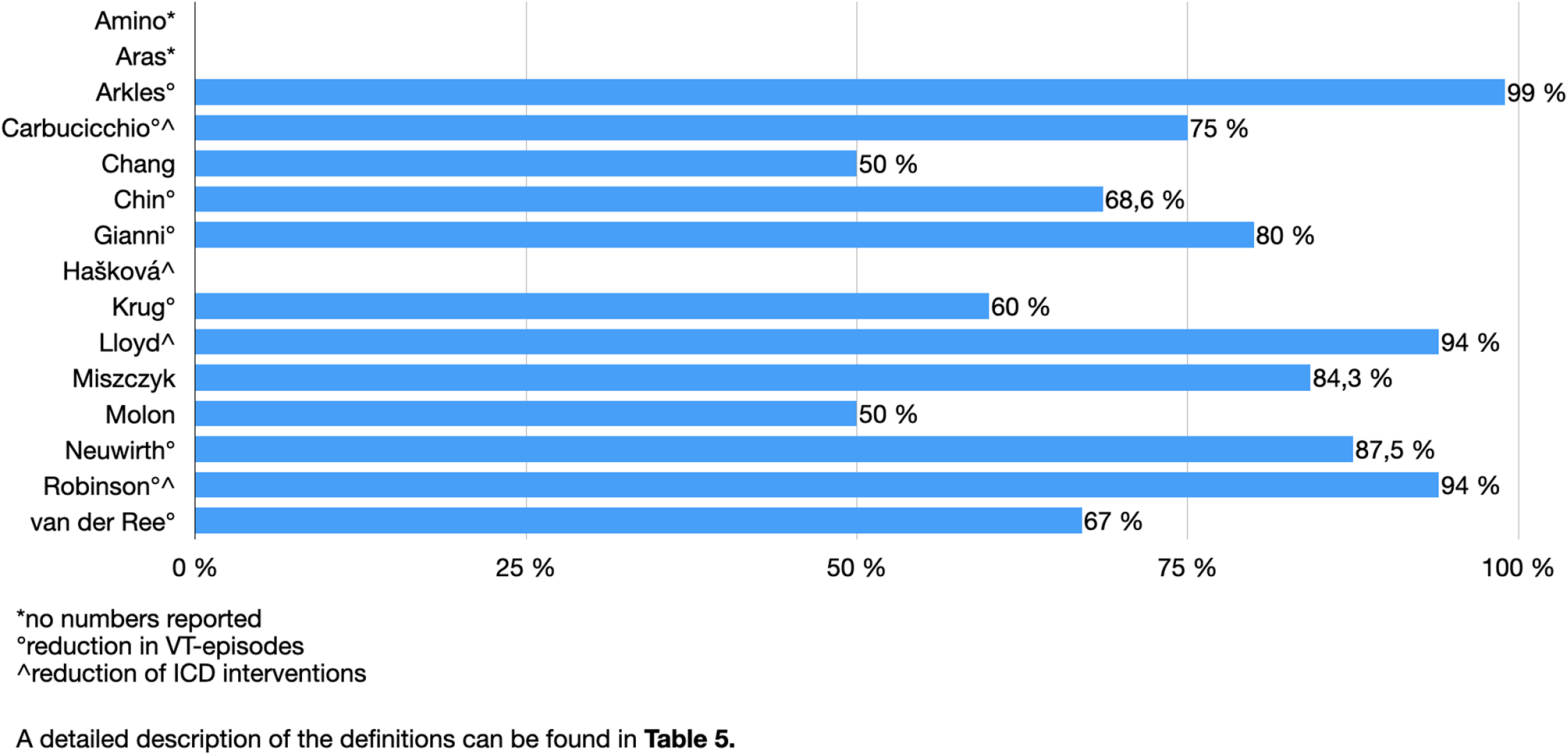
VT-Reduction

A detailed overview about VT-burden and VT-reduction is shown in Table 5 and Fig. 2.

**Table 5:** VT-Reduction and follow-up overview

Author	Definition of VT Burden	VT Reduction	Planned Follow up (months)	Follow-up (months); mean / median / range	Additional Findings
Amino et al.	ICD therapies	Median reduction (not quantified)	not reported	not reported/ not reported / 6 - 30	AAD dose reduction in 3/5 patients; 1 patient remained VT-free
Aras et al.	VT episodes, ICD shocks, ATP	Reduction in all parameters	12	not reported / 8 / 1 - 14	VT recurrence in 5/6 after 3 months; 1 remained shock- and ATP-free at 6 months
Arkles et al.	Treated VT episodes	99% reduction	12	not reported	no further interventions required
Carbucicchio et al.	VT episodes, ICD interventions	Reduction in 3/4 patients	12	not reported / 8 / not reported	not reported
Chang et al.	VT recurrence	Complete remission in 3/6 patients	12	not reported	no need for salvage therapy
Chin et al.	VT episodes (median)	Decrease from 35 to 11 episodes	12	not reported / 7.8 / not reported	reduction not statistically significant
Gianni et al.	VT episodes	4/5 with initial reduction; 1 with increased episodes	18	12 ± 2 / not reported / 10 - 14	AADs restarted in all; 3 underwent repeat ablation
Hašková et al.	ICD therapies	Significant reduction in DC shocks	not reported	not reported / 26.9 / not reported	47% underwent repeat ablation due to VT recurrence
Krug et al.	VT episodes	Reduction in 3/5 patients	60	not reported	not reported
Lloyd et al.	ICD therapies	69% reduction (94% excluding 1 non-responder)	not reported	not reported	not reported
Miszczyk et al.	VT burden (not clearly defined)	84.3% reduction	not reported	not reported / 22.2 / 1.3 - 28.6	not reported
Molon et al.	Freedom from VT burden	3/6 patients remained VT-free	not reported	not reported	not reported
Neuwirth et al.	VT episodes	87.5% reduction	12	not reported / 28 / 16 - 54	not reported
Robinson et al.	VT episodes, ICD shocks	94% reduction; 69% had VT recurrence post-blanking	12	not reported	decrease in ICD shocks and AAD use
van der Ree et al.	Treated VT episodes	≥50% reduction in 67% of patients	12	not reported / not reported / 7 - 12	not reported

#### Mortality

Mortality was mentioned in 14 of 15 studies. The range of total mortality reached from 0% [13] to 51% (*n* = 18) during the follow up [12]. However, a substantial proportion of reported deaths were not directly attributed to radiotherapy. In several studies, the cause of death was either reported as unrelated, unlikely to be related or remained uncertain. Of the 51 mentioned events, 26 were cardiogenic causes, consisting of progressive heart failure (*n* = 24) and cardiogenic shock (*n* = 2). The remaining 25 events were not of cardiogenic origin and varied significantly.

**Fig. 3:**
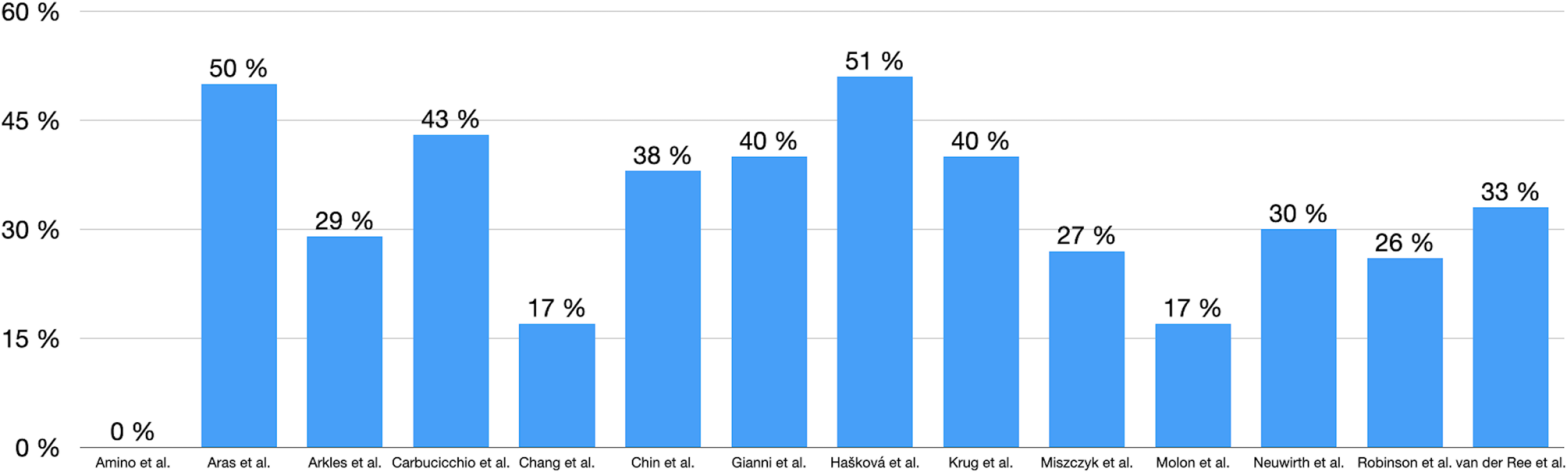
Mortality

A detailed overview is presented in Table 6, and a graphical representation of the mortality is shown in Fig. 3.

**Table 6:** Mortality

Author	*N*	Deaths	Total mortality	Unrelated to treatment	Causes of death
Amino et al.	3	0	0%	not reported	not reported
Aras et al.	8	4	50%	3	COVID-19 sepsis (2) gastric cancer (1) progressive heart failure with recurrent VT (1)
Arkles et al.	15	4	29%	not reported	aspiration pneumonia (1) cardiogenic shock (1) cause undetermined (1) progressive heart (RV) failure (1)
Carbucicchio et al.	8	3	43%	2	progressive heart failure (1) multiorgan failure following bacterial pneumonia and sepsis (1) cause undeterminded (1)
Chang et al.	6	1	17%	1	pneumonia (1)
Chin et al.	8	3	38%	2	multiple organ failure after perforated appendix (1) sepsis of unclear etiology and multiorgan failure (1) progressive heart failure (1)
Gianni et al.	6	2	40%	not reported	progressive of heart failure (2)
Hašková et al.	36	18	51%	not reported	progressive heart failure (12) sudden death during recurrence of myocardial infarction (1) sudden unwitnessed death (1) COVID-19 pneumonia (1) pneumonia after stroke (1) carcinoma (1) bleeding caused by esophago-pericardial fistula (1)
Krug et al.	5	2	40%	not reported	cardiogenic shock (1) progressive heart failure (1)
Lloyd et al.	10	not reported	not reported	not reported	not reported
Miszczyk et al.	11	3	27%	not reported	advanced ovarian cancer (1) acute heart transplant rejection (1) progressive heart failure, pneumonia, and sepsis (1)
Molon et al.	6	1	17%	not reported	progressive heart failure (1)
Neuwirth et al.	10	3	30%	1	vascular dementia; Alzheimer’s disease (1) progressive heart failure (2)
Robinson et al.	19	5	26%	1	progressive heart failure (1) VT/VF while driving (1) accidential asphyxiation (1) amiodarone pulmonary toxicity (1) Biliary stent complicated by pancreatitis, sepsis, respiratory failure, renal failure; withdrew care (1)
van der Ree et al.	6	2	33%	2	aspiration (1) progressive respiratory failure (1)

#### Quality of life

QoL was only mentioned in 4 of 15 studies, with 3 of them [15, 19, 21] using the SF-36 questionnaire and one [20] utilizing the EQ-5D questionnaire.

In the study by Carbucicchio et al. [17], the SF-36 questionnaire demonstrated a slight overall improvement at 6 months in physical functioning (16 to 35), role limitations due to physical health (25 to 38), health perception (37 to 52), and vitality (34 to 53), while no consistent changes were observed in the mental component summary. Concurrently, Robinson et al. [25] reported improvements in 5 of 9 SF-36 domains at 6 months, with no declines in any domain. Van der Ree et al. [21] found QoL improvements in 8 of 9 domains (89%) at 12 months (*n* = 4), with both summary scale scores (physical and mentally) increasing. A median improvement of + 68% (range: +21 to + 121) was observed in the mental component summary, while the physical component summary exhibited a median increase of + 13% (range: −6 to + 27). Additionally, Miszczyk et al. [15] assessed QoL using the EQ-5D questionnaire and found improvements in 5 out of 8 evaluable patients at 3 months, 5 out of 7 at 6 months, and 4 out of 7 at 12 months.

### Safety of cardiac stereotactic body radiation

A total of 11 out of the 15 studies referenced AEs. In 10 of these studies the AEs were classified using the Common Terminology Criteria for Adverse Events (CTCAE) version 4.0 or 5.0. Most of the AEs were classified as mild to moderate, but there were also 4 Grade 5 events mentioned [12, 22]. The one study not employing CTCAE reported pericardial effusions in 2 patients (1 asymptomatic, 1 resolved with medical management). Apart from this, no definitive organ toxicities were detected during a median follow-up period of 8 months [11].

Acute toxicity was documented in only 3 of the 15 studies, with the following events recorded: one patient exhibited slow VT below the treatment zone of the patient’s device during SBRT [14]; 4 patients experienced nausea [16] and 1 patient demonstrated a radiation therapy-induced electrical reset of the ICD [21].

Only 6/15 studies documented the constraint guidelines that were used. Among these, 3 studies [14, 19, 21] employed the TG-101 guideline, while RAVENTA [18] and SMART-VT [15] established their own limits. Notably, 1 study [22] utilized lung SBRT constraints.

A comprehensive summary of all AEs with a grade ≥ 3 CTCAE rating, both before and after 90 days, is provided in Table 7.

**Table 7:** Adverse events with CTCAE grade ≥ 3

Author	<90 d				>90 d				Constraint guidelines
	Adverse events (*n*)	CTCAE (*n* / *N* (%))			Adverse events (*n*)	CTCAE (*n* / *N* (%))			
		Grade 3	Grade 4	Grade 5		Grade 3	Grade 4	Grade 5	
**Amino et al.**					-heart failure	1 / 3 (33.3)			
**Aras et al.**					-death			1 / 8 (12.5)	
**Arkles et al.**					-death			3 / 14 (21.4)	Lung constraints for SBRT
**Carbucicchio et al.**									
**Chang et al.**					-heart failure	1 / 6 (16.7)			
**Chin et al.**									
**Gianni et al.**									TG-101
**Hašková et al.**					-death (1) -progression of mitral regurgitation (3)	3 / 36 (8.3)	1 / 36 (2.8)		
**Krug et al.**									RAVENTA
**Lloyd et al.**									TG-101
**Miszczyk et al.**	-heart failure		1 / 11 (9.0)						SMART-VT
**Molon et al.**									
**Neuwirth et al.**					-progression of mitral regurgiation (1) -heart failure (1) -pericarditis (1)	3 / 10 (30.0)			
**Robinson et al.**	-heart failure (1) -pericarditis (1)	2 / 19 (10.5)							
**van der Ree et al.**					-chest wall pain	1 / 6 (16.7)			TG-101

## Discussion

CA is considered standard-of-care for drug-refractory VT in structural heart disease. However, it has limitations such as incomplete lesion formation, procedural risks, and inaccessible substrates [26].

In this context, SBRT has emerged as a potential adjunctive or salvage option, especially in patients with elevated procedural risk for invasive catheterization.

Stereotactic body radiation therapy has commonly been utilized in radiation oncology for the treatment of solid tumors. While initially restricted to the brain, technical and planning progresses have extended its application to further areas throughout the body including moving targets, such as liver, lung– and even small areas within the beating heart [5]. Furthermore, SBRT was found to reduce the direct and total procedural costs compared with VT ablation by 49% and 54%, respectively [27].

It is important to acknowledge that the patient populations included in these studies typically represent a high-risk group with complex clinical profiles. ICM was observed in over half of the patients in most cohorts, reflecting the advanced structural heart disease underlying VT. The proportion of patients with prior catheter ablation varied widely, highlighting a significant number of cases where SBRT was applied as a salvage or adjunctive therapy. The reported NYHA classes ranged from I to IV, with a predominance of class II and III, and LVEF was generally reduced. These characteristics underscore the complexity and severity of the disease in the patients treated with SBRT, providing essential context for interpreting the outcomes and safety data presented in subsequent sections.

Treatments were delivered with standard LINAC or Cyberknife^®^ devices, with VMAT being the predominant delivery technique. The use of advanced field modulation techniques, such as VMAT or sliding-window IMRT, appears essential to accommodate the complexities of a moving target, ensuring adequate dose conformity despite cardiac and respiratory motion. The treatment planning was basically comparable with SBRT planning for malignant diseases. However, specialized EAM and motion management techniques were usually applied in order to correctly identify small ectopic areas within the heart and compensate for displacements by heartbeat and breathing.

Given the technical challenges and the evolving evidence base, SBRT for VT should preferably be delivered within clinical trials or in highly specialized centers with structured interdisciplinary protocols. An interdisciplinary framework must ensure close collaboration with cardiologists to support structured patient selection, precise treatment planning, and thorough post-procedural monitoring. The underlying techniques are largely transferable to routine radiation oncology practice. However, widespread clinical adoption should await further validation through controlled studies and the development of consensus-based guidelines, particularly regarding target volume definition. In the future, SBRT may offer a widely accessible and cost-effective adjunct or alternative to CA in selected patients, particularly in centers with established collaboration between cardiac electrophysiology and radiation oncology.

This systematic review demonstrates that SBRT is associated with a considerable reduction in VT burden across first studies. With total VT-reduction rates ranging from 50 to 99%, the data diverged, but a decline was clearly perceived. There are no direct comparisons between SBRT and CA yet. A meta-analysis found CA reduced VT recurrence by 18% and ICD shocks by 38% versus medical therapy [28]. In another meta-analysis, SBRT reduced VT recurrence by 90% and ICD shocks by 91%. but differences in study populations and the absence of a control group limit conclusions [7]. Nevertheless, a pivotal, multicenter, randomized trial, RADIATE-VT (NCT05765175), is currently ongoing. This trial compares the safety and efficacy of SBRT with that of repeat CA in patients diagnosed with high-risk, refractory VT. The primary completion of the RADIATE-VT study is projected in May 2026.

While most of the studies reported sustained benefits, a few others showed, that VT recurrence remains a problem for some patients [16, 25, 26]. This discrepancy may be attributable to variations in patient selection criteria, targeting and treatment characteristic and follow-up duration. This points out the need for additional standardized trials to determine long-term efficacy and safety.

Unfortunately, QoL was only assessed in a few studies. The results reached from moderate improvements [17, 25] to valuable achievements, particularly in the mental component [21]. Miszczyk et al. [15] further supported these findings with incremental increase in QoL over time. These findings suggest the potential benefit of SBRT.

Mortality was reported in nearly all studies, with significant variation in causes, including both cardiovascular and non-cardiovascular diseases. In a pooled analysis by Benali et al. [29], a high 1-year mortality rate of 32% was found, with over one-third of deaths occurring within the first three months post-SBRT, though no clear association with SBRT parameters was identified. The primary causes were progressive heart failure and non-cardiac conditions, aligning with the studies in this review. Severe complications were unlikely, with four documented Grade 5 events [12, 22] and rare acute toxicity such as slow VT below the treatment zone of the patient’s device during SBRT, nausea and radiation therapy-induced electrical reset of the ICD [14, 16, 21].

The findings of this study show variability in toxicity across constraint guidelines. The TG-101 studies [14, 19, 21] and the RAVENTA-study [18] reported no or mild toxicities, while SMART-VT [15] noted grade 4 heart failure and the lung SBRT constraint study [22] reported three late deaths. Due to the small number of studies reporting detailed toxicity data and the differences in patient populations, it is difficult to draw firm conclusions when comparing toxicities across various constraint guidelines. Additionally, variations in imaging, protocols, and follow-up further limit comparability between studies.

According to the current guidelines [30], routine monitoring of ICDs during radiotherapy is generally recommended to promptly identify and manage potential device malfunctions. However, such AEs are rare, and in cases where low-dose normofractionated radiotherapy is administered away from cardiac target volumes, the necessity for intensive monitoring may be reduced [31, 32]. However, due to the mostly single-arm design and lack of control groups, it is not possible to distinguish this worsening of cardiac function from the natural course of the disease. In contrast, Dukkipati et al. [33] described in a review about CA that serious procedure-related complications may occur in 7% of the patients. These complications include cardiac perforation (1.5%), major vascular injury (2.3%), and death (up to 3%). Other complications that may appear are uncontrollable VT (up to 2.6%), stroke or transient ischemic attack (0.5%), heart block (0.9%), and coronary artery injury (0.2%). Furthermore, acute hemodynamic decompensation was documented in 11% of the patients and is associated with an elevated mortality rate. Severe complications related to the invasive character of cardiac catheterization are not to be expected due to the nature of external beam radiotherapy. This underscores the potential benefits of SBRT as a less invasive adjunct or alternative. Beyond that, SBRT only requires a minimum in-patient treatment duration, favoring its application in elderly and frail patients [34], who are at elevated risk for secondary AEs, such as delirium, nosocomial infections and embolic events [35]. Given the overall treatment period of < 60 min in most of the patients, SBRT is at least comparable to CA in terms of treatment duration.

A significant challenge in the treatment of VT with SBRT lies in the precise delineation of the target volume. The various imaging modalities employed for scar identification, such as cardiac PET-CT or EAM, possess limitations that introduce uncertainty in target definition and complicating comparisons across SBRT protocols. Recent preclinical data from a porcine infarct model demonstrated that a single 25 Gy fraction significantly reduced VT inducibility and resulted in denser scar formation compared to untreated controls [36]. These findings suggest that there is functional modulation of the arrhythmogenic substrate without extensive fibrosis in remote myocardium. While these results support the efficacy of a 25 Gy dose in this model, they also suggest that higher doses are likely required for complete scar deactivation. Given the yet unclear optimum dose and fractionation schedule further investigation is warranted in order to reduce normal tissue damage.

Despite the promising results, the most relevant limitation of the current evidence according to the literature available is the absence of randomized controlled trials, what makes it difficult to establish causality. Admittedly, conducting a randomized trial in such a vulnerable patient population is likely to be associated with substantial logistical and ethical challenges. Additionally, variations in patient selection criteria, VT definition, endpoint assessment and follow-up duration restrict comparability of the results.

## Conclusion

SBRT appears to be an effective and safe non-invasive treatment to reduce VT burden within first clinical trials. However, it is currently best considered as a complementary or salvage option in patients who have undergone prior CA procedures, rather than as a primary alternative. To date, evidence is limited to small, non-controlled cohorts, and SBRT can currently not be considered as a routine treatment. The delivery of SBRT should be administered in a clinical trial setting or within specialized medical centers that have established interdisciplinary protocols. Future prospective phase-(II)/III trials with standardized endpoints are needed to confirm the long-term efficacy and safety. Treatment delivery is basically comparable with SBRT for malignant diseases but requires specialized imaging and mapping procedures to correctly identify and ensure precise radiation delivery on small ectopic areas within the beating heart.

## Supplementary Information

Below is the link to the electronic supplementary material.

Supplementary Material 1

## Data Availability

No datasets were generated or analysed during the current study.

## Abbreviations

AADs: antiarrhythmic drugs
AE: adverse events
ATP: anti-tachycardia pacing
CA: catheter ablation
CPAP: continuous positive airway pressure
CBCT: cone beam CT
CTCAE: Common Terminology Criteria for Adverse Events
DCA: dynamic conformal arc
DIBH: deep inspiration breath-hold
EAM: electroanatomic mapping
ICD: implantable cardioverter-defibrillator
ICM: ischemic cardiomyopathy
IMAT: intensity-modulated arc therapy
ITV: internal target volume
LVEF: left ventricular ejection fraction
NYHA: New York Heart Association
PTV: planning target volume
QoL: Quality of life
SABR: stereotactic ablative body radiotherapy
SBRT: Stereotactic body radiation therapy
STAR: stereotactic arrhythmia radioablation
VMAT: volumetric modulated arc therapy
VT: ventricular tachycardia

## References

[1] Foth C, Gangwani MK, Ahmed I, Alvey H. Ventricular tachycardia. StatPearls. Treasure Island (FL). Volume 30. StatPearls Publishing; 2023. 30422549

[2] Martinek M, Manninger M, Schönbauer R, Scherr D, Schukro C, Pürerfellner H, et al. Expert consensus on acute management of ventricular arrhythmias - VT network Austria. Int J Cardiol Heart Vasc. 2021;34:100760. 10.1016/j.ijcha.2021.100760. 33869728 10.1016/j.ijcha.2021.100760 PMC8047164

[3] Looi KL, Tang A, Agarwal S. Ventricular arrhythmia storm in the era of implantable cardioverter-defibrillator. Postgrad Med J. 2015;91(1079):519–26. 10.1136/postgradmedj-2015-133550. 26310265 10.1136/postgradmedj-2015-133550

[4] Liu G, Xu X, Yi Q, Lv T. The efficacy of catheter ablation versus ICD for prevention of ventricular tachycardia in patients with ischemic heart disease: a systematic review and meta-analysis. J Interv Card Electrophysiol. 2021;61(3):435–43. 10.1007/s10840-020-00848-1. 33723693 10.1007/s10840-020-00848-1 PMC8376706

[5] Wei C, Qian PC, Boeck M, Bredfeldt JS, Blankstein R, Tedrow UB, et al. Cardiac stereotactic body radiation therapy for ventricular tachycardia: current experience and technical gaps. J Cardiovasc Electrophysiol. 2021;32(11):2901–14. 10.1111/jce.15259. 34587335 10.1111/jce.15259

[6] Benedict SH, Yenice KM, Followill D, Galvin JM, Hinson W, Kavanagh B, et al. Stereotactic body radiation therapy: the report of AAPM task group 101. Med Phys. 2010;37:4078–101. 10.1118/1.3438081. 20879569 10.1118/1.3438081

[7] Gupta A, Sattar Z, Chaaban N, Ranka S, Carlson C, Sami F, et al. Stereotactic cardiac radiotherapy for refractory ventricular tachycardia in structural heart disease patients: a systematic review. Europace. 2024;27(1):euae305. 10.1093/europace/euae305. 39716963 10.1093/europace/euae305 PMC11780863

[8] Miszczyk M, Hoeksema WF, Kuna K, Blamek S, Cuculich PS, Grehn M, et al. Stereotactic arrhythmia radioablation (STAR)-A systematic review and meta-analysis of prospective trials on behalf of the stopstorm.eu consortium. Heart Rhythm. 2025;22(1):80–9. 10.1016/j.hrthm.2024.07.029. 39032525 10.1016/j.hrthm.2024.07.029

[9] Page MJ, McKenzie JE, Bossuyt PM, Boutron I, Hoffmann TC, Mulrow CD, et al. The PRISMA 2020 statement: an updated guideline for reporting systematic reviews. BMJ. 2021;372:n71. 10.1136/bmj.n71. Published 2021 Mar 29. 33782057 10.1136/bmj.n71 PMC8005924

[10] Higgins JPT, Thomas J, Chandler J, Cumpston M, Li T, Page MJ, Welch VA, editors. *Cochrane Handbook for Systematic Reviews of Interventions* version 6.5 (updated August 2024). Cochrane, 2024. Available from www.training.cochrane.org/handbook

[11] Aras D, Çetin EHÖ, Ozturk HF, Ozdemir E, Kara M, Ekizler FA, et al. Stereotactic body radioablation therapy as an immediate and early term antiarrhythmic palliative therapeutic choice in patients with refractory ventricular tachycardia. J Interv Card Electrophysiol. 2023;66(1):135–43. 10.1007/s10840-022-01352-4. 36040658 10.1007/s10840-022-01352-4 PMC9424800

[12] Hašková J, Wichterle D, Kautzner J, Šramko M, Peichl P, Knybel PEng L, et al. Efficacy and safety of stereotactic radiotherapy in patients with recurrent ventricular tachycardias: the Czech experience. JACC Clin Electrophysiol. 2024;10(4):654–66. 10.1016/j.jacep.2023.12.002. 38385912 10.1016/j.jacep.2023.12.002

[13] Amino M, Kabuki S, Kunieda E, Hashimoto J, Sugawara A, Sakai T, et al. Interim report of a Japanese phase II trial for cardiac stereotactic body radiotherapy in refractory ventricular Tachycardia - Focus on target determination. Circ Rep. 2023;5(3):69–79. 10.1253/circrep.CR-23-0003. Published 2023 Feb 9. 36909137 10.1253/circrep.CR-23-0003 PMC9992511

[14] Lloyd MS, Wight J, Schneider F, Hoskins M, Attia T, Escott C Clinical experience of stereotactic body radiation for refractory ventricular tachycardia in advanced heart failure patients. Heart Rhythm et al. 2020;17(3):415–422. 10.1016/j.hrthm.2019.09.028 10.1016/j.hrthm.2019.09.028 31585181

[15] Miszczyk M, Sajdok M, Bednarek J, Latusek T, Wojakowski W, Tomasik B et al. Stereotactic management of arrhythmia- radiosurgery in treatment of ventricular tachycardia (SMART-VT). Results of a prospective safety trial. Radiother Oncol. 2023;188. 10.1016/j.radonc.2023.109857 37597807

[16] Neuwirth R, Cvek J, Knybel L, Jiravsky O, Molenda L, Kodaj M, et al. Stereotactic radiosurgery for ablation of ventricular tachycardia. Europace. 2019;21(7):1088–95. 10.1093/europace/euz133. 31121018 10.1093/europace/euz133

[17] Carbucicchio C, Andreini D, Piperno G, Catto V, Conte E, Cattani F, et al. Stereotactic radioablation for the treatment of ventricular tachycardia: preliminary data and insights from the STRA-MI-VT phase ib/ii study. J Interventional Cardiac Electrophysiol. 2021;62(2):427–39. 10.1007/s10840-021-01060-5. 10.1007/s10840-021-01060-5 PMC8490832 34609691

[18] Krug D, Zaman A, Eidinger L, Grehn M, Boda-Heggemann J, Rudic B, et al. Radiosurgery for ventricular tachycardia (RAVENTA): interim analysis of a multicenter multiplatform feasibility trial. Strahlenther Onkol. 2023;199(7):621–30. 10.1007/s00066-023-02091-9. 37285038 10.1007/s00066-023-02091-9 PMC10245341

[19] Gianni C, Rivera D, Burkhardt JD, Pollard B, Gardner E, Maguire P, et al. Stereotactic arrhythmia radioablation for refractory scar-related ventricular tachycardia. Heart Rhythm. 2020;17(8):1241–8. 10.1016/j.hrthm.2020.02.036. 32151737 10.1016/j.hrthm.2020.02.036

[20] Chang WI, Jo HH, Cha MJ, Chang JH, Choi CH, Kim HJ, et al. Short-term and long-term effects of noninvasive cardiac radioablation for ventricular tachycardia: A single-center case series. Heart Rhythm O2. 2023;4(2):119–26. 10.1016/j.hroo.2022.11.006. 36873313 10.1016/j.hroo.2022.11.006 PMC9975004

[21] van der Ree MH, Dieleman EMT, Visser J, Planken RN, Boekholdt SM, de Bruin-Bon RHA, et al. Non-invasive stereotactic arrhythmia radiotherapy for ventricular tachycardia: results of the prospective STARNL-1 trial. Europace. 2023;25(3):1015–24. 10.1093/europace/euad020. 36746553 10.1093/europace/euad020 PMC10062344

[22] Arkles J, Markman T, Trevillian R, Yegya-Raman N, Garg L, Nazarian S, et al. One-year outcomes after stereotactic body radiotherapy for refractory ventricular tachycardia. Heart Rhythm. 2024;21:18–24. 10.1016/j.hrthm.2023.10.005. 37827346 10.1016/j.hrthm.2023.10.005

[23] Chin R, Hayase J, Hu P, Cao M, Deng J, Ajijola O, et al. Non-invasive stereotactic body radiation therapy for refractory ventricular arrhythmias: an institutional experience. J Interventional Cardiac Electrophysiol. 2021;61(3):535–43. 10.1007/s10840-020-00849-0. 10.1007/s10840-020-00849-0 32803639

[24] Molon G, Giaj-Levra N, Costa A, Bonapace S, Cuccia F, Marinelli A, et al. Stereotactic ablative radiotherapy in patients with refractory ventricular tachyarrhythmia. Eur Heart J Supplement. 2022;24:C248–53. 10.1093/eurheartj/suac016. 10.1093/eurheartj/suac016 PMC9117912 35602256

[25] Robinson CG, Samson PP, Moore KMS, Hugo GD, Knutson N, Mutic S, et al. Phase I/II trial of Electrophysiology-Guided noninvasive cardiac radioablation for ventricular tachycardia. Circulation. 2019;139(3):313–21. 10.1161/CIRCULATIONAHA.118.038261. 30586734 10.1161/CIRCULATIONAHA.118.038261 PMC6331281

[26] Cuculich PS, Schill MR, Kashani R, Mutic S, Lang A, Cooper D, Faddis M, Gleva M, Noheria A, Smith TW, Hallahan D, Rudy Y, Robinson CG. Noninvasive cardiac radiation for ablation of ventricular tachycardia. N Engl J Med. 2017;377(24):2325–36. 10.1056/NEJMoa1613773. 29236642 10.1056/NEJMoa1613773 PMC5764179

[27] Wei C, Boeck M, Qian PC, Vivenzio T, Elizee Z, Bredfeldt JS, et al. Cost of cardiac stereotactic body radioablation therapy versus catheter ablation for treatment of ventricular tachycardia. Pacing Clin Electrophysiol. 2022;45(9):1124–31. 10.1111/pace.14512. 35621224 10.1111/pace.14512

[28] Virk SA, Kumar S. Catheter ablation of ventricular tachycardia in patients with structural heart disease: a meta-analysis. JACC Clin Electrophysiol. 2023;9:255–7. 10.1016/j.jacep.2022.09.002. 36858693 10.1016/j.jacep.2022.09.002

[29] Benali K, Zei PC, Lloyd M, Kautzner J, Guenancia C, Ninni S, et al. One-year mortality and causes of death after stereotactic radiation therapy for refractory ventricular arrhythmias: A systematic review and pooled analysis. Trends Cardiovasc Med. 2024;34(7):488–96. 10.1016/j.tcm.2023.12.008. 38191005 10.1016/j.tcm.2023.12.008

[30] Gauter-Fleckenstein B, Israel CW, Dorenkamp M, Dunst J, Roser M, Schimpf R, et al. DEGRO/DGK guideline for radiotherapy in patients with cardiac implantable electronic devices. Strahlenther Onkol. 2015;191(5):393–404. 10.1007/s00066-015-0817-3. 25739476 10.1007/s00066-015-0817-3

[31] Warmbrunn J, Straube C, Haase HU, Sinnecker D, Laugwitz KL, Combs SE, et al. Influence of radiotherapy on cardiac implantable devices and leads-a single-institution analysis and critical evaluation of current guidelines. Strahlenther Onkol. 2025;201(4):463–71. 10.1007/s00066-024-02345-0. 39792262 10.1007/s00066-024-02345-0 PMC11928412

[32] Steger F, Hautmann MG, Süß C, Hubauer U, Ücer E, Maier L, et al. Radiotherapy of patients with cardiac implantable electronic devices according to the DEGRO/DGK guideline-is the risk of relevant errors overestimated? Radiotherapie von patienten Mit kardialen implantierbaren elektronischen geräten entsprechend der DEGRO/DGK-Leitlinie– wird Das Risiko für relevante fehlfunktionen überschätzt? Strahlenther Onkol. 2019;195(12):1086–93. 10.1007/s00066-019-01502-0. 31399799 10.1007/s00066-019-01502-0

[33] Dukkipati SR, Koruth JS, Choudry S, Miller MA, Whang W, Reddy VY. Catheter ablation of ventricular tachycardia in structural heart disease: indications, strategies, and Outcomes-Part II. J Am Coll Cardiol. 2017;70(23):2924–41. 10.1016/j.jacc.2017.10.030. 29216988 10.1016/j.jacc.2017.10.030

[34] Cuccia F, Mazzola R, Pastorello E, Figlia V, Giaj-Levra N, Nicosia L, et al. SBRT for elderly oligometastatic patients as a feasible, safe and effective treatment opportunity. Clin Exp Metastasis. 2021;38(5):475–81. 10.1007/s10585-021-10122-x. 34487288 10.1007/s10585-021-10122-x

[35] Szlejf C, Farfel JM, Curiati JA, Couto Ede B Jr, Jacob-Filho W, Azevedo RS. Medical adverse events in elderly hospitalized patients: a prospective study. Clin (Sao Paulo). 2012;67(11):1247–52. 10.6061/clinics/2012(11)04. 10.6061/clinics/2012(11)04 PMC3488980 23184198

[36] Kancharla K, Olson A, Salavatian S, Kuwabara Y, Martynyuk Y, Dutta P, et al. Ventricular arrhythmia inducibility in Porcine infarct model after stereotactic body radiation therapy. Heart Rhythm. 2024;21(7):1154–60. 10.1016/j.hrthm.2024.02.037. 38395245 10.1016/j.hrthm.2024.02.037

