# Supplementary material for: Technical aspects of SBRT for therapy-refractory ventricular tachycardia: a systematic review for radiation oncologists
Source: Radiat Oncol. 2025 Aug 29;20:136. doi: 10.1186/s13014-025-02704-w (PMC12395866; doi:10.1186/s13014-025-02704-w)
Supplement: Supplementary file 1 — Supplementary Material 1 [file 13014_2025_2704_MOESM1_ESM.docx]

**Technical aspects of SBRT for therapy-refractory ventricular tachycardia: A systematic review for radiation oncologists**

**Appendix**

**A1 Search string**

**Pubmed**

(("Tachycardia, Ventricular"[Mesh] OR "Tachycardia, Ventricular/drug therapy"[Mesh] OR "Tachycardia, Ventricular/economics"[Mesh] OR "Tachycardia, Ventricular/radiotherapy"[Mesh] OR "Tachycardia, Ventricular/therapy"[Mesh]) OR (ventricular tachycardia[Text Word]))

AND

(("Radiotherapy"[Mesh] OR "Radiotherapy/adverse effects"[Mesh] OR "Radiotherapy/economics"[Mesh] OR "Radiotherapy/statistics and numerical data"[Mesh]) OR (“Radiotherapy” [Text Word])

OR

("Radiosurgery"[Mesh] OR "Radiosurgery/adverse effects"[Mesh] OR "Radiosurgery/economics"[Mesh] OR "Radiosurgery/statistics and numerical data"[Mesh] ) OR (“Radiosurgery” [Text Word])

OR

("Radiation"[Mesh]) OR ("Radiation"[Text word]))

### 510 results, 01/2025

**Scopus**

( TITLE-ABS-KEY ( ventricular  AND tachycardia )  OR  TITLE-ABS-KEY ( tachycardia,  AND ventricular  AND  therapy )  OR  TITLE-ABS-KEY ( tachycardia,ventricular  AND  radiotherapy )  OR  TITLE-ABS-KEY ( tachycardia,ventricular  AND  drug  AND therapy )  OR  TITLE-ABS-KEY ( tachycardia,ventricular  AND  economics )  AND  TITLE-ABS-KEY ( radiotherapy )  OR  TITLE-ABS-KEY ( radiotherapy  AND  adverse  AND effects )  OR  TITLE-ABS-KEY ( radiotherapy  AND  economics )  OR  TITLE-ABS-KEY ( radiotherapy  AND  statistics,  AND numerical,  AND data )  OR  TITLE-ABS-KEY ( radiosurgery )  OR  TITLE-ABS-KEY ( radiosurgery  AND  adverse  AND effects )  OR  TITLE-ABS-KEY ( radiosurgery  AND  economics )  OR  TITLE-ABS-KEY ( radiosurgery  AND  statistics,  AND numerical,  AND data )  OR  TITLE-ABS-KEY ( radiation )  OR  TITLE-ABS-KEY ( radiation  AND  adverse  AND effects )  OR  TITLE-ABS-KEY ( radiation  AND  economics )  OR  TITLE-ABS-KEY ( radiation  AND  statistics,  AND numerical,  AND data ) )

### 998 results, 01/2025

**Cochrane**

(tachycardia ventricular):ti,ab,kw

OR (tachycardia ventricular therapy):ti,ab,kw

OR (Tachycardia,ventricular radiotherapy):ti,ab,kw

OR (Tachycardia,ventricular drug therapy):ti,ab,kw

OR (Tachycardia,ventricular economics):ti,ab,kw

AND

((radiotherapy):ti,ab,kw

OR (radiotherapy adverse effects):ti,ab,kw

OR (radiotherapy economics):ti,ab,kw

OR (radiotherapy statistics and numerical data):ti,ab,kw

OR

(radiosurgery):ti,ab,kw

OR (radiosurgery adverse effects):ti,ab,kw

OR (radiosurgery economics):ti,ab,kw

OR

(radiation):ti,ab,kw

OR (radiation adverse effects):ti,ab,kw

OR (radiation economics):ti,ab,kw )

### 3212 results, 01/2025

**Web of Science**

(TS=(“tachycardia ventricular*”)

OR TS=(“tachycardia ventricular* therapy”)

OR TS=(“Tachycardia,ventricular* radiotherapy”)

OR TS=(“Tachycardia,ventricular* radio therapy”)

OR TS=(“Tachycardia,ventricular* drug therapy”)

OR TS=(“Tachycardia,ventricular* drugtherapy”)

OR TS=(“Tachycardia,ventricular* economics”))

AND

(TS=(“radiotherapy*”) OR TS= (“radio therapy*”)

OR TS=(“radiotherapy adverse effects*”)

OR TS=(“radio therapy adverse effects*”)

OR TS=(“radiotherapy economics*”)

OR TS=(“radio therapy economics*”)

OR TS=(“radiotherapy statistics and numerical data*”)

OR TS=(“radio therapy statistics and numerical data*”)

OR TS=(“radiotherapy statistics*”)

OR TS=(“radio therapy statistics*”)

OR TS=(“radiotherapy numerical data*”)

OR TS=(“radio therapy numerical data*”)

OR

TS=(“radiosurgery*”)

OR TS=(“radiosurgery adverse effects*”)

OR TS=(“radiosurgery economics*”) OR TS=(“radio surgery adverse effects*”)

OR TS=(“radio surgery economics*”)

OR

TS=(“radiation treatment*”)

OR TS=(“radiation treatment adverse effects*”)

OR TS=(“radiation treatment economics*”)

OR

TS=(“radiation*”)

OR TS=(“radiation adverse effects*”)

OR TS=(“radiation economics*”)))

### 1678 results, 01/2025

**A2 reasons for excluding studies**

| **Year** | **Author** | **Title** | **Reason for excluding** |
| --- | --- | --- | --- |
| 2020 | Blanck et al. | Radiosurgery for ventricular tachycardia: preclinical and clinical evidence and study design for a German multi-center multi-platform feasibility trial (RAVENTA) | Study protocol, no results provided |
| 2021 | Carbucicchio et al. | STRA-MI-VT (STereotactic RadioAblation by Multimodal Imaging for Ventricular Tachycardia): rationale and design of an Italian experimental prospective study | Study protocol, no results provided |
| 2023 | Cellini et al. | Ventricular tachycardia ablation through radiation therapy (VT-ART) consortium: Concept description of an observational multicentric trial via matched pair analysis | Study protocol, no results provided |
| 2020 | Kiani et al. | Histopathologic and Ultrastructural Findings in Human Myocardium After Stereotactic Body Radiation Therapy for Recalcitrant Ventricular Tachycardia | Study on explanted hearts |
| 2019 | Knutson et al. | Radiation Therapy Workflow and Dosimetric Analysis from a Phase 1/2 Trial of Noninvasive Cardiac Radioablation for Ventricular Tachycardia | Overlapping data with ENCORE-VT trial |
| 2023 | Kovacs et al. | Dose escalation for stereotactic arrhythmia radioablation of recurrent ventricular tachyarrhythmia - a phase II clinical trial | Treatment technique different per PICO criteria (dose escalation trial) |
| 2021 | Krug et al. | Recommendations regarding cardiac stereotactic body radiotherapy for treatment refractory ventricular tachycardia | Overlapping data with RAVENTA trial |
| 2020 | Krug et al. | Stereotactic body radiotherapy for ventricular tachycardia (cardiac radiosurgery) : First-in-patient treatment in Germany | Overlapping data with RAVENTA trial |
| 2023 | Mayinger et al. | Quality assurance process within the RAdiosurgery for VENtricular TAchycardia (RAVENTA) trial for the fusion of electroanatomical mapping and radiotherapy planning imaging data in cardiac radioablation | Only quality control of the technology and overlapping data with the RAVENTA trial |
| 2021 | Miszczyk et al. | Stereotactic management of arrhythmia – radiosurgery in treatment of ventricular tachycardia (SMART-VT) – clinical trial protocol and study rationale | Overlapping data with SMART-VT |
| 2021 | Reis et al. | SBRT of ventricular tachycardia using 4pi optimized trajectories | Treatment technique different per PICO criteria |

**A3 Risk of Bias**

| **Author** | **Bias due to confounding** | **Bias in selection of participants** | **Bias in classification of interventions** | **Bias due to deviations from intended interventions** | **Bias due to missing data** | **Bias in measurement of outcomes** | **Bias in selection of reported results** | **Evidence level (Oxford)** |
| --- | --- | --- | --- | --- | --- | --- | --- | --- |
| Amino et al. | high | moderate | low | moderate | moderate | moderate | high | 4 |
| Aras et al. | high | moderate | low | moderate | moderate | moderate | high | 4 |
| Arkles et al. | high | moderate | low | moderate | moderate | moderate | high | 4 |
| Carbucicchio et al. | high | moderate | low | moderate | moderate | moderate | high | 4 |
| Chang et al. | high | moderate | low | moderate | moderate | moderate | high | 4 |
| Chin et al. | high | moderate | low | moderate | moderate | moderate | high | 4 |
| Gianni et al. | high | moderate | low | moderate | moderate | moderate | high | 4 |
| Hašková et al. | high | moderate | low | moderate | moderate | moderate | high | 4 |
| Krug et al. | high | moderate | low | moderate | moderate | moderate | high | 4 |
| Lloyd et al. | high | moderate | low | moderate | moderate | moderate | high | 4 |
| Miszczyk et al. | high | moderate | low | moderate | moderate | moderate | high | 4 |
| Molon et al. | high | moderate | low | moderate | moderate | moderate | high | 4 |
| Neuwirth et al. | high | moderate | low | moderate | moderate | moderate | high | 4 |
| Robinson et al. | high | moderate | low | moderate | moderate | moderate | high | 4 |
| van der Ree et al. | high | moderate | low | moderate | moderate | moderate | high | 4 |

**A 4 Techniques for Motion Control, Planning, and Post-Therapy Monitoring**

| **Author** | **Motion management** | **Treatment planning/ contouring of the target volume** | **Post-treatment measures** |
| --- | --- | --- | --- |
| Amino et al. | vacuum-fixed cushion, abdominal compression technique | Target delineation: electrical (12-lead ECG, intracardiac electrophysiology study), structural (electroanatomic voltage mapping, cardiac MRI, thoracic CT, echocardiography), and functional (scintigraphy with 99mTc-tetrofosmin, 123I-MIBG, 123I-BMIPP) data were integrated, substrates/foci (ON) and non-target regions (OFF) were identified using a 17-segment model, organs at risk were contoured  Dose calculation: Performed using the Acuros XB algorithm  PTV margin : 2 - 5 mm | vital sign assessment, ICD checks, blood and biochemical data, ECG, high-resolution ambulatory ECG, chest CT (as appropriate) |
| Aras et al. | average phase CT for cardiac and respiratory movements,  ITV was created by expanding CTV | Target delineation: 4D-CT, PET-CT, EAM; internal gross target volume was defined by merging PET-CT and avgCT images, identifying hypometabolic scar regions  PTV margin: 5 mm | oral anticoagulation |
| Arkles et al. | Abdominal compression devices, 4D contrast CT-based simulation planning, Isocenter was marked using the free breathing scan, ITV was created by expanding GTV | Target delineation: 3D-EAM (87%), cardiac CT angiography (93%), cardiac MRI (80%), F18 PET (33%), manual registration aligned EAM with anatomical landmarks (aortic cusps, LV apex, mitral annulus)  PTV margin: 3 mm | ECG, remote monitoring |
| Carbucicchio et al. | 4D CT, ICD marker for respiratory motion | Target delineation: cardiac CT, EAM, ECG, and ECGI data  Free-breathing CT: identified CTV via semi-automatic fibrosis localization, along with organs at risk and the ICD  4D-CT: assessed cardiac and respiratory motion, expanding the CTV to ITV  PTV margin: not specified | routine laboratory tests and echocardiography examination  (24 h after SBRT), 12-lead ECG and ICD interrogation, echocardiography examination, thorax CT  (at 3 and 12 months) |
| Chang et al. | ITV delineation using CPAP (n=1), deep-inspiration breath-hold DIBH (n=2), and 4D CT | Target delineation: synthesis of imaging studies, 12-lead ECG, EAM, chest CT, cardiac MRI, and single-photon emission CT   PTV margin: 5 mm | ECG with 24-hour monitoring or telemetry  (before and after treatment), 12-lead ECG  (the day before SBRT, the day of SBRT, 1 week and 1 month post-SBRT) |
| Chin et al. | whole-body vacuum bag (Body-FIX, Elekta, Stockholm, Sweden), CT simulation scan, 4DCT,  monitoring with ECG telemetry and peripheral pulse oximetry during the treatment | Target delineation: wideband late gadolinium enhancement (LGE) MRI for scar characterization, and EAM , organs at risk were contoured  PTV margin: 6 - 8 mm | repeat trans-thoracic echocardiography  (3 months after SBRT) |
| Gianni et al. | ITV: CTV expanded by 3 mm, Synchrony Respiratory Tracking for real-time motion compensation, fiducial marker placed near the PTV to the motion of the chest wall | Target delineation: cardiac CT, EAM, and 12-lead ECG; temporary transjugular pacing lead served as a fiducial marker for tracking respiratory motion; organs at risk were contoured   PTV margin: not specified | ECG |
| Hašková et al. | ITV for cardiac motion, breath-hold CT, ICD marker for respiratory motion | Target delineation: breath-hold contrast CT, ITV-based, CTV by EAM–CT co-registration  PTV margin: 0 - 5 mm | ICD interrogation and echocardiography  (every 6 months), chest x-ray  (when clinically indicated) |
| Krug et al. | DIBH (n=1) , ITV (n=4); cardiac internal target volume (CITV) accounted for cardiac motion; ICD marker for respiratory motion | Target delineation: EAM and contrast-enhanced, ECG-gated cardiac CT, including the arrhythmogenic substrate  PTV margin: 3 - 5 mm | monitoring  (after treatment) |
| Lloyd et al. | 4D CT; ICD marker for respiratory motion | Target delineation: 3D imaging , EAM, incorporating recurrent VT morphology, remaining inducible VTs post-ablation, organs at risk were contoured with dose constraints following TG101 guidelines.  PTV margin: 1 - 5 mm | not reported |
| Miszczyk et al. | DIBH (n=10) or free-breathing respiratory gating (n=1); continuous surface-guided (cardiac monitoring) | Target delineation: EAM, 12-lead ECG, ICD memory readouts, prior CA records, and cardiac-gated contrast-enhanced CT, organs at risk, coronary artery sparing  PTV margin: 3 mm | 12- lead ECG, Holter ECG |
| Molon et al. | 4D-CT scans (3-mm slices); abdominal thermoplastic mask for immobilization | Target delineation: EAM and imaging data, PET-CT, organs at risk  PTV margin: 5 mm | not reported |
| Neuwirth et al. | ITV created from expiratory breath-hold CT with contrast and ECG-gated scans (systole/diastole); ICD marker for respiratory motion | Target delineation: 3D-EAM, accounting for cardiac motion  PTV margin: not specified | transthoracic echocardiography,  ICD interrogation |
| Robinson et al. | vacuum-assisted device or foam cushion coupled with an abdominal compression device | Target delineation: cardiac CT, MRI, PET-CT, 12-lead ECG, and ECG imaging during induced VT via noninvasive programmed stimulation  PTV margin: 5 mm | ECG, Holter ECG, CT scans, and ECGI  (at day 3; weeks 2, 4, and 6; months 3, 6, and 12; then annually) |
| van der Ree et al. | heart rhythm monitoring; 4D-CT; ITV: based on 4D-CT data | Target delineation: EAM and imaging data, 12-lead ECGs, invasive electrophysiology study maps, ECG, CT, nuclear imaging, and MRI   PTV margin: 5 mm | 24 h telemetric observation, 12-lead ECGs, laboratory tests, ICD readout, echocardiography |
